# Functional Outcomes and Predictors of Response in Knee Osteoarthritis Following a Home-Based Structured Exercise Program

**DOI:** 10.7759/cureus.107428

**Published:** 2026-04-20

**Authors:** Semi H Maden, Asem R Chanu, Srikumar Venkataraman, Chandan J Das, Bangkim C Khangembam

**Affiliations:** 1 Physical Medicine and Rehabilitation, All India Institute of Medical Sciences, New Delhi, IND; 2 Radiodiagnosis and Interventional Radiology, All India Institute of Medical Sciences, New Delhi, IND; 3 Nuclear Medicine, All India Institute of Medical Sciences, New Delhi, IND

**Keywords:** clinical outcome, functional outcome, home-based exercise, knee osteoarthritis, predictors of response

## Abstract

Background

Structured exercise is a key conservative approach in managing knee osteoarthritis, but the effectiveness of home-based programs and predictors of individual response remain less explored. The study aimed to assess the effectiveness of a structured home-based exercise program on functional outcomes in knee osteoarthritis (OA) and identify predictors of successful outcome.

Methods

This prospective longitudinal study included 30 patients with knee OA who followed an eight-week home-based exercise regimen involving warm-up, strengthening, stretching, and neuromuscular control exercises. Functional outcomes were assessed using six-minute walk test (6MWT) and Knee Injury and Osteoarthritis Outcome Score (KOOS) at baseline, four weeks, and eight weeks. Baseline imaging included radiographs and three-phase bone scan with single-photon emission computed tomography-computed tomography (SPECT/CT). A successful clinical outcome was defined as Patient Global Impression of Change score <3 at eight weeks.

Results

Participants (age: 50±7 years; 25 women) showed significant improvement in 6MWT (P<0.001) and total KOOS (P=0.025), particularly in the sports and recreation subdomain (P=0.022). Sixty percent (18/30) of patients achieved a successful outcome. Early KOOS improvement at four weeks was the only independent predictor of successful clinical outcome (odds ratio (OR): 1.115; P=0.047). Imaging parameters, including SPECT/CT, did not predict outcome.

Conclusion

Home-based exercise significantly improved function in knee OA. Early changes in KOOS scores predicted outcome, while imaging findings were not predictive.

## Introduction

Knee osteoarthritis (OA) is a highly prevalent degenerative joint disease and a leading cause of chronic pain and functional limitation among older adults globally [1,2]. It imposes a considerable burden on patients’ quality of life, healthcare systems, and society. Non-pharmacological approaches such as physical therapy and structured exercise programs have emerged as first-line options alongside pharmacologic approaches, especially in early-to-moderate stages of OA [3,4]. Among exercise regimens, there is no established hierarchy; however, supervised exercises have shown better outcomes compared to unsupervised regimens [5,6]. However, practical barriers - including cost, time constraints, and limited facility access - often prevent regular supervised sessions. This necessitates structured home-based programs as scalable alternatives. Despite their potential, the effectiveness of home-based programs and predictors of clinical outcome remain insufficiently explored.

Outcome assessment in OA commonly relies on patient-reported measures like the Western Ontario and McMaster Universities Osteoarthritis Index (WOMAC) and the Knee Injury and Osteoarthritis Outcome Score (KOOS) [7,8]. While these tools capture subjective experiences of pain, symptoms, and functionality, objective functional tests such as the Six-Minute Walk Test (6MWT) quantitatively evaluate mobility and endurance [9]. Integrating both subjective and objective assessments offers a holistic view of a patient’s clinical outcome.

Identifying predictors of clinical outcome is critical for personalized management in knee OA. Early clinical indicators could enable timely management modifications, while imaging parameters might refine patient selection. However, the role of imaging in predicting conservative management outcomes remains unclear. Imaging modalities like three-phase bone scan with single-photon emission computed tomography-computed tomography (SPECT/CT) add another layer of understanding by detecting functional information localized to specific compartments over and above the structural changes visible on standard radiographs [10,11]. This modality can be proposed as a tool to assess soft tissue inflammation and compartment-specific subchondral activity and thereby explore potential imaging predictors of clinical outcome. This study aimed to evaluate the effectiveness of a structured home-based exercise program in knee OA and identify clinical and imaging-based predictors of successful outcome. The primary objective was to assess changes in the 6MWT at four and eight weeks. Secondary objectives included evaluating changes in KOOS and its subdomains, and identifying predictors of successful clinical outcome at eight weeks.

## Materials and methods

This prospective longitudinal study was conducted over two years from August 2022 to July 2024. The Institute Ethics Committee for Post-Graduate Research approved the study (IECPG-547/28.07.2022), and all patients provided signed written informed consent. The study adhered to the ethical standards of the Declaration of Helsinki 1964, revised in 1983, and its subsequent amendments. The study matches all the items of the Transparent Reporting of Evaluations with Nonrandomized Designs (TREND) statement [12].

Sample size calculation

The sample size was determined using STATA 17.0 (StataCorp LLC, College Station, TX) based on an anticipated mean difference of 40 meters between baseline and follow-up values of 6MWT, with a standard deviation of 75 meters, as reported in a prior study evaluating home-based exercise programs in knee OA [13]. Assuming a study power of 80% and a two-sided significance level of 5%, the required sample size was calculated to be 28 participants. To account for a potential 10% dropout rate, the final target sample size was set at 30 participants.

Patients

Thirty consecutive adult patients (age >40 years) diagnosed with knee OA as per the American College of Rheumatology criteria [14] were prospectively recruited. Participants were excluded if they had a history of recent knee interventions, coexisting joint disorders, lower limb fractures or surgeries, neurological conditions, cognitive impairments, or any medical conditions that contraindicated performing the 6MWT. Participants were recruited through physician referrals from the outpatient services of the Department of Physical Medicine and Rehabilitation of a tertiary care academic medical institute. No systematic sampling plan was implemented; recruitment was based on consecutive sampling. Clinical assessments were conducted at the outpatient facility, while imaging was performed in the hospital’s Radiology and Nuclear Medicine departments.

Home-based structured exercise program

Participants were instructed to perform a structured home-based exercise program that included warm-up, strengthening, stretching, and neuromuscular control components, with periodic progression. Participants were provided with exercise pamphlets detailing the structured regimen. Those identified as overweight or obese were encouraged to pursue weight reduction strategies. Additionally, joint protection techniques were recommended as part of the conservative management approach. Alongside the exercise program, participants were permitted to use analgesic medications on an as-needed basis, specifically etoricoxib 60 mg or diclofenac up to 150 mg/day under a proton pump inhibitor cover. Exercises were to be discontinued if the participant experienced an increase in pain of ≥2 points on the Numerical Pain Rating Scale (NPRS) [15]. The exercise program was structured into three daily sessions, each comprising the components as detailed in Table 1.

**Table 1:** Home-based structured exercise program reps: repetitions, sec: seconds, min: minutes

Component	Exercise Type	Level / Timeline	Description	Dosage
Warm-up	Walking	Baseline onward	Walking on flat surface	5 min
Strengthening	Quadriceps	Level 1a (Baseline)	Isometric contraction (long sitting)	10 reps, 3-sec hold
		Level 1b (Week 1)	Seated knee extension (full range)	10 reps, 3-sec hold
		Level 2 (Week 2)	Seated knee extension+250 g weights	10 reps, 3-sec hold
		Level 3 (Week 4)	Seated knee extension+500 g weights	10 reps, 3-sec hold
	Hip Extensors	Throughout	Pelvic bridging (knees 90°)	10 reps, 3-sec hold
	Hamstrings	Level 1 (Baseline)	Prone knee flexion (0-90°)	10 reps, 3-sec hold
		Level 2 (Week 2)	Prone knee flexion+250 g weights	10 reps, 3-sec hold
		Level 3 (Week 4)	Prone knee flexion+500 g weights	10 reps, 3-sec hold
Stretching	Achilles	Throughout	Standing lunge stretch	10 sec hold, total 2 min
	Hamstrings	Throughout	Seated forward reach	10 sec hold, total 2 min
Neuromuscular Control	Weight shifts	Level 1 (Baseline)	Lateral and forward shifts	3×30 sec (10 sec break in between), total 2 min
		Level 2 (Week 4)	+ tandem stance shifts	3×30 sec (10 sec break in between), total 2 min

Assessment

In addition to a comprehensive clinical evaluation, all participants underwent baseline imaging that included anteroposterior radiographs of the knee in weight-bearing position to assess the tibiofemoral compartment, as well as skyline views for evaluating the patellofemoral compartment, along with a three-phase bone scan with SPECT/CT. Functional outcome measures included the 6MWT and KOOS [16, 17]. Grading on radiographs was recorded based on Kellgren-Lawrence (KL) and Sperner classifications [18, 19]. Radiotracer uptake on the three-phase bone scan was evaluated during the flow, blood pool, and delayed static phases, with documentation of compartments exhibiting increased activity on SPECT/CT. The specialists interpreting the imaging studies were aware of the clinical diagnosis but were blinded to all other clinical details. To ensure adherence and monitor progress, telephonic follow-up calls were conducted at one and two weeks. Compliance was assessed through structured telephonic follow-up and participant self-report, rather than objective monitoring devices (e.g., wearable devices or digital tracking systems). Follow-up assessments were conducted at four and eight weeks in the outpatient clinic, during which the outcome measures were reassessed. A successful clinical outcome was defined as a Patient Global Impression of Change (PGIC) score of <3 at eight weeks [20,21]. The PGIC score is a validated single-item patient-reported outcome measure that assesses the patient’s overall perception of improvement since the start of the management. It uses a seven-point ordinal scale ranging from 1 (‘very much improved’) to 7 (‘very much worse’), with 4 indicating ‘no change.’ A score of < 3 (i.e., 1 and 2 indicating ‘very much improved’ and ‘much improved’ respectively) was considered indicative of a successful clinical outcome.

Statistical analysis

Descriptive statistics were employed to summarize the data, including mean±standard deviation (SD), median (range), frequencies, and percentages. The distribution of quantitative variables was assessed using the Shapiro-Wilk test for normality. Changes in quantitative outcomes across the three time points - baseline, four weeks, and eight weeks - were analyzed using repeated measures analysis of variance (ANOVA) with Greenhouse-Geisser correction to account for sphericity violations. Post-hoc pairwise comparisons between time points were conducted with Bonferroni correction to control for multiple comparisons. To identify independent predictors of clinical outcomes, both univariate and multivariate logistic regression analyses were performed. A two-tailed P value <0.05 was considered indicative of statistical significance. All statistical analyses were performed with statistical packages STATA 17.0, IBM SPSS 26 (IBM Corp., Somers, NY, USA), MedCalc® 20.009 (Med-Calc Software, Mariakerke, Belgium), and XLSTAT 2022.5.1 (Addinsoft Inc., New York, USA).

## Results

Patient characteristics

The study cohort had a mean age of 50±7 years, with a predominantly female population, accounting for 83.3% (25/30) of the participants. Based on structured telephonic follow-ups at weeks one and two, and physical follow-ups at weeks four and eight, high levels of self-reported adherence were observed, with no reported episodes of significant pain exacerbation (≥2 points on the NPRS) during the study period. A substantial proportion of the participants (96.7%; 29/30) had a sedentary occupation [22], and the majority (80%; 24/30) presented with clinically symptomatic bilateral knee OA. Radiographic evaluation based on the Kellgren-Lawrence (KL) grading system revealed that most participants were categorized as grade 2 or 3, indicating mild to moderate structural disease severity. Furthermore, 66.7% (20/30) of the participants demonstrated prominent radiotracer uptake in the tibiofemoral compartment on baseline SPECT/CT imaging, consistent with active joint involvement and increased subchondral bone turnover in the affected region. A detailed representation of patient characteristics is shown in Table 2.

**Table 2:** Patient characteristics *In case of bilateral knee involvement, higher grade was taken. #In case of bilateral knee involvement, the side with higher uptake was taken. SD: standard deviation, KL grading: Kellgren-Lawrence grading, SPECT/CT: single-photon emission computed tomography-computed tomography, PGIC: Patient Global Impression of Change score

Characteristics	Value
Age (years)	
Mean±SD	50±7
Median (range)	50 (41-69)
Sex	
Male	5 (16.7%)
Female	25 (83.3%)
Body mass index (kg/m^2^)	
Mean±SD	29.8±4.2
Median (range)	30.7 (21.4-39.3)
Duration of symptoms (months)	
Mean±SD	53.4±57.6
Median (range)	30.0 (1.0-240.0)
KL grading^*^	
Grade 1	8 (26.7%)
Grade 2	11 (36.7%)
Grade 3	9 (30.0%)
Grade 4	2 (6.6%)
Sperner grading^*^	
Grade 1	15 (50.0%)
Grade 2	11 (36.7%)
Grade 3	4 (13.3%)
Education	
Below matriculation	14 (46.7%)
Matriculation or higher	16 (53.3%)
Attendant	
Available	13 (43.3%)
Not available	17 (56.7%)
Marital status	
Unmarried	2 (6.7%)
Married	28 (93.3%)
Comorbidity	
Absent	18 (60.0%)
Present	12 (40.0%)
Occupation	
Sedentary	29 (96.7%)
Non-sedentary	1 (3.3%)
Symptomatic limb	
Unilateral	6 (20.0%)
Bilateral	24 (80.0%)
Deformity	
Absent	14 (46.7%)
Present	16 (53.3%)
Past intervention	
Absent	24 (80.0%)
Present	6 (20.0%)
Analgesic medication	
No	16 (53.3%)
Yes	14 (46.7%)
Three-phase bone scan with SPECT/CT	
Laterality	
Unilateral	3 (10.0%)
Bilateral	27 (90.0%)
Flow-pool uptake	
Negative	10 (33.3%)
Positive (one limb)	8 (26.7%)
Positive (both limbs)	12 (40.0%)
Most prominent side	
Left knee	18 (60.0%)
Right knee	12 (40.0%)
Compartments involved^#^	
<3 compartments	21 (70.0%)
3 compartments	9 (30.0%)
Most prominent compartment	
Patello-femoral	10 (33.3%)
Tibio-femoral	20 (66.7%)
Uptake in other joints of lower limb	
Absent	15 (50.0%)
Contralateral	3 (10.0%)
Bilateral	12 (40.0%)
Clinical outcome	
Unsuccessful (PGIC Score ≥3)	12 (40.0%)
Successful (PGIC Score <3)	18 (60.0%)

Functional improvement

A significant improvement in 6MWT performance was observed over the study period. The mean distance increased from 322.45±59.80 meters at baseline to 346.13±56.35 meters at four weeks, and further to 361.36±63.70 meters at eight weeks, with the overall change reaching statistical significance (P<0.001). Post-hoc pairwise comparisons between each time point also demonstrated statistically significant differences (P≤0.009) (Table 3).

**Table 3:** Functional parameters across different time points ANOVA: analysis of variance, 6MWT: six-minute walk test, KOOS: Knee Injury and Osteoarthritis Outcome Score, η²ₚ: partial eta squared, ADL: activities of daily living. *P value significant at <0.05 level.

Parameter	Baseline	4 weeks	8 weeks	Repeated measures ANOVA			Post-hoc pairwise comparison with Bonferroni correction					
				F	η²ₚ	P value	4 weeks vs baseline		8 weeks vs 4 weeks		8 weeks vs baseline	
							Mean difference	P value	Mean difference	P value	Mean difference	P value
6MWT (m)	322.45±59.80	346.13±56.35	361.36±63.70	21.752	0.429	<0.001^*^	23.68	0.002^*^	15.23	0.009^*^	38.91	<0.001^*^
KOOS	46.23±16.81	47.63±16.70	52.10±16.53	4.205	0.127	0.025^*^	1.40	1.000	4.467	0.077	5.867	0.077
Subdomains of KOOS												
Symptoms	57.53±18.43	59.97±17.92	63.87±19.89	4.316	0.130	0.024^*^	2.43	0.604	3.90	0.190	6.33	0.061
Pain	55.07±18.72	55.57±17.93	57.90±18.99	0.710	0.024	0.471	0.50	1.000	2.33	0.910	2.83	1.000
ADL	53.07±19.06	53.97±19.86	58.70±19.95	2.409	0.077	0.112	0.90	1.000	4.73	0.078	5.63	0.286
Sports and recreation	30.00±22.01	35.67±23.15	39.97±23.67	5.648	0.163	0.008^*^	5.67	0.111	4.30	0.409	9.97	0.022^*^
Knee-related quality of life	34.40± 21.51	32.97±15.60	36.77±16.20	0.620	0.021	0.493	1.433	1.000	-3.80	0.402	-2.37	1.000

The KOOS total score exhibited a statistically significant overall improvement, rising from 46.23±16.81 at baseline to 52.10±16.53 at eight weeks (P=0.025). Post-hoc pairwise comparisons between individual time points revealed a trend toward statistical significance in improvements from four weeks to eight weeks, and from baseline to eight weeks (P=0.077). Among the KOOS subdomains, both symptoms and sports and recreation showed significant overall improvement over time (P≤0.024). However, on post-hoc pairwise comparisons, only the sports and recreation subdomain exhibited a statistically significant improvement from baseline to eight weeks (P=0.022), while the symptoms subdomain demonstrated a trend toward significance over the same period (P=0.061) (Table 3).

Predictors of successful outcome

A successful clinical outcome at eight weeks, defined as a PGIC score <3, was achieved by 60% (18/30) of participants. Univariate analysis identified the change in the total KOOS score at four weeks as a significant predictor of successful clinical outcome. This association remained significant on multivariate analysis, where the change in total KOOS score at four weeks emerged as an independent predictor of a favourable outcome (odds ratio: 1.115; 95% Confidence Interval: 1.002-1.241; P=0.047) (Table 4).

**Table 4:** Predictors of clinical outcome 95% CI: 95% confidence interval, BMI: body mass index, KL grading: Kellgren-Lawrence grading, ADL: activities of daily living, KOOS: Knee Injury and Osteoarthritis Outcome score. *P value significant at <0.05 level.

	Univariate analysis				Multivariate analysis			
Parameter	B	Odd’s ratio	95% CI	P value	B	Odd’s ratio	95% CI	P value
Age	-0.007	0.993	0.886-1.112	0.899				
Sex	-1.145	0.318	0.031-3.268	0.335				
BMI	-0.029	0.972	0.814-1.160	0.751				
Duration of symptoms	0.013	1.013	0.995-1.031	0.156	0.015	1.016	0.992-1.039	0.191
KL grading	0.544	1.722	0.720-4.119	0.222				
Sperner grading	-0.111	0.895	0.319-2.509	0.833				
Education	-0.223	0.800	0.185-3.460	0.765				
Attendant	-1.030	0.357	0.079-1.615	0.181	0.310	1.364	0.209-8.901	0.746
Marital status	-0.435	0.647	0.037-11.454	0.767				
Comorbidity	0.693	2.000	0.448-8.936	0.364				
Symptomatic limb	-0.511	0.600	0.099-3.634	0.578				
Deformity	-0.223	0.800	0.185-3.460	0.765				
Past intervention	0.511	1.667	0.275-10.094	0.578				
Analgesic medication	-0.336	0.714	0.164-3.117	0.654				
Laterality on bone scan	-1.224	0.294	0.024-3.671	0.342				
Flow-pool uptake on bone scan	-0.230	0.794	0.335 – 1.884	0.601				
Most prominent side on bone scan	-0.470	0.625	0.137-2.852	0.544				
Compartments involved on bone scan	-1.157	0.314	0.052-1.882	0.205				
Most prominent compartment on bone scan	-0.619	0.538	0.115-2.519	0.432				
Uptake in other joints of lower limb on bone scan	-0.345	0.708	0.324-1.548	0.387				
Change in 6MWT at 4 weeks	-0.005	0.995	0.973-1.017	0.662				
Change in KOOS at 4 weeks	0.096	1.101	1.000-1.211	0.049^*^	0.109	1.115	1.002-1.241	0.047^*^

## Discussion

This prospective study highlights the effectiveness of a structured home-based exercise program in improving functional outcomes in patients with knee OA. The significant increase in 6MWT distance at four and eight weeks demonstrates the program’s utility in enhancing endurance and mobility, two key metrics in OA rehabilitation [5]. Importantly, this improvement was achieved in a population where over 80% presented with bilateral knee symptoms and a sedentary occupation, suggesting the feasibility of implementing such approaches in real-world settings with limited resources or access to supervised care [1]. The magnitude of functional improvement observed in the present study is comparable to that reported in prior studies evaluating home-based exercise programs for knee OA. Deyle et al. demonstrated that a home exercise program consisting of strengthening, stretching, and range-of-motion exercises led to an approximate 10% improvement in 6MWT distance (~36 meters) at four weeks in a cohort of 68 participants with knee OA [13]. In the current study, participants achieved a similar pattern of improvement, with a 7.3% increase (~24 meters) at four weeks and a more pronounced 12.1% increase (~39 meters) at eight weeks. Notably, the baseline functional capacity in the present cohort was lower (mean 6MWT distance: 322 meters vs 408 meters), suggesting a more functionally limited population at enrolment. Despite this, the absolute improvement at eight weeks was comparable, if not slightly greater, which may reflect the added benefit of neuromuscular control exercises incorporated into the current program. Furthermore, while Deyle et al. reported a 12% dropout rate over eight weeks, the present study observed no dropouts, which may indicate good tolerability and feasibility of the home-based regimen in the studied population, though this finding should be interpreted cautiously, given the reliance on self-reported adherence. These convergent findings across studies support the robustness and reproducibility of home-based exercise programs as an effective conservative management strategy for knee OA.

The overall improvement in total KOOS and select subdomains, particularly symptoms and sports and recreation, indicates a positive shift in self-perceived functionality and quality of life. However, the lack of significant change in the pain and knee-related quality of life subdomains underscores a complex pain profile in OA patients that may not be fully addressed by exercise alone. This aligns with the growing understanding that OA pain is multifactorial, often encompassing biomechanical, neuropathic, and psychosocial components [23]. Therefore, exercise approaches might benefit more from integration of other non-pharmacological (patient education, weight management, assistive devices, activity modification, cognitive-behavioural therapy) and pharmacological adjuncts for a more holistic approach.

A notable finding of the study is the role of early changes in total KOOS score as a predictor of successful clinical outcome at eight weeks, indicating that early improvements in patient-reported outcomes can guide clinicians in decision-making and therapy modulation. In the current study, body mass index (BMI) did not appear to significantly impact the extent of functional improvement achieved through the home-based exercise program. This finding diverges from earlier studies that have commonly linked higher BMI with increased pain, reduced physical function, and diminished clinical outcome in individuals with knee OA [24, 25]. Although obesity remains a well-recognized contributor to OA onset and progression, this study indicates that BMI may not pose a substantial barrier to functional gains when exercise approaches are structured and adherence is high. This aligns with evidence suggesting that individuals across a range of BMI levels can experience similar benefits from exercise, especially when these approaches are personalized and engagement is sustained [26]. It is worth noting that the average BMI of the current study participants (29.8±4.2 kg/m²) was comparatively lower than in many previous investigations, which may partially account for the differing outcomes observed. Collectively, these findings support the implementation of exercise programs irrespective of BMI, emphasizing patient participation and consistent progression in overweight status alone.

In this study, no imaging parameters derived from three-phase bone scan with SPECT/CT predicted clinical outcomes. While prior studies have linked subchondral bone activity to OA progression and surgical outcomes [10,11], the absence of such associations in this study suggests that functional improvement following conservative management may be more influenced by modifiable behavioural factors, such as physical activity levels and adherence to exercise, than by baseline imaging findings. Although SPECT/CT remains valuable for assessing joint activity and subchondral bone turnover, its predictive utility for non-surgical management outcomes appears limited, consistent with mixed evidence in the literature [5,6]. This raises questions about routine SPECT/CT use in guiding conservative management of early-stage knee OA, despite its recognized role in surgical planning [10, 11]. However, as three-phase bone scan with SPECT/CT was employed in an exploratory capacity in this study, and given the limited sample size, these results should be interpreted with caution. Further large-scale studies are required to better define its role in guiding conservative management strategies in early-stage knee OA. Also, the lack of a strong correlation between structural imaging findings on knee radiographs and clinical improvement suggests that, in early-stage knee OA managed conservatively, functional recovery may be independent of structural disease severity. As a result, performance-based measures like the 6MWT and patient-reported outcomes such as the KOOS may offer greater clinical relevance by more accurately capturing real-world disability and therapeutic progress.

This study has several strengths. The prospective longitudinal design with structured follow-up enabled systematic assessment of temporal functional changes. Integration of objective (6MWT) and subjective (KOOS) measures provided a comprehensive evaluation of clinical outcome. The exploratory use of three-phase bone scan with SPECT/CT to assess advanced imaging-based predictors, while yielding null findings, contributes valuable information for future research design. The exercise program incorporated neuromuscular training alongside traditional exercises, reflecting contemporary evidence-based practice. Finally, a high adherence rate suggests good feasibility and acceptability, supporting potential real-world implementation.

Despite its strengths, the study is not free from limitations. The small sample size (N=30) limits external validity and statistical power, especially for subgroup analyses. The short eight-week follow-up may miss long-term positive outcomes or relapses. Furthermore, the reliance on patient self-reporting for KOOS and PGIC introduces subjectivity and potential recall bias. The absence of objective adherence monitoring (e.g., wearable or digital tracking tools) represents an important limitation and may affect the reliability of compliance-related findings. Future studies incorporating wearable technologies could offer more accurate adherence tracking and individualized feedback loops.

In terms of future research, expanding the cohort size and extending the follow-up duration would help determine the sustainability of benefits and identify late responders. Comparative studies between home-based and supervised approaches, potentially blended with tele-rehabilitation platforms, are warranted. Additionally, exploring the incorporation of dietary modifications, weight management strategies, and behavioural therapy may further improve outcomes.

## Conclusions

This prospective study demonstrates that a structured home-based exercise program can significantly improve functional mobility and patient-reported outcomes in individuals with knee osteoarthritis over an eight-week period. Early improvement in total KOOS score at four weeks emerged as an independent predictor of successful clinical outcome, suggesting that early monitoring of patient-reported functional changes may help guide management decisions and identify responders. Although imaging modalities, including three-phase bone scan with SPECT/CT, did not predict short-term functional outcomes in this cohort, the small sample size limits definitive conclusions, highlighting the need for larger multicenter prospective studies.
